# The role of helminths in the development of non-communicable diseases

**DOI:** 10.3389/fimmu.2022.941977

**Published:** 2022-08-31

**Authors:** Yifan Wu, Megan Duffey, Saira Elizabeth Alex, Charlie Suarez-Reyes, Eva H. Clark, Jill E. Weatherhead

**Affiliations:** ^1^ Department of Pediatrics, Division of Tropical Medicine, Baylor College of Medicine, Houston, TX, United States; ^2^ Department of Medicine, Section of Infectious Diseases, Baylor College of Medicine, Houston, TX, United States; ^3^ National School of Tropical Medicine, Baylor College of Medicine, Houston, TX, United States

**Keywords:** helminths, non-communicable diseases, cestodes, trematodes, nematodes, immunity

## Abstract

Non-communicable diseases (NCDs) like cardiovascular disease, chronic respiratory diseases, cancers, diabetes, and neuropsychiatric diseases cause significant global morbidity and mortality which disproportionately affect those living in low resource regions including low- and middle-income countries (LMICs). In order to reduce NCD morbidity and mortality in LMIC it is imperative to understand risk factors associated with the development of NCDs. Certain infections are known risk factors for many NCDs. Several parasitic helminth infections, which occur most commonly in LMICs, have been identified as potential drivers of NCDs in parasite-endemic regions. Though understudied, the impact of helminth infections on the development of NCDs is likely related to helminth-specific factors, including species, developmental stage and disease burden. Mechanical and chemical damage induced by the helminth in combination with pathologic host immune responses contribute to the long-term inflammation that increases risk for NCD development. Robust studies from animal models and human clinical trials are needed to understand the immunologic mechanisms of helminth-induced NCDs. Understanding the complex connection between helminths and NCDs will aid in targeted public health programs to reduce helminth-induced NCDs and reduce the high rates of morbidity that affects millions of people living in parasite-endemic, LMICs globally.

## The global impact of non-communicable diseases

Non-communicable diseases (NCDs) such as cardiovascular disease, chronic respiratory diseases, cancers, diabetes, and neuropsychiatric diseases are now the most common causes of morbidity and mortality globally, including in low- and middle-income countries (LMICs) (1). NCDs are responsible for over 71% of all deaths worldwide and 1.6 billion disability adjusted life years (DALYs) (2). The rise in NCDs is likely multifactorial due to changes in lifestyle including reduced physical activity and non-nutritious diets, increased ability to diagnose NCDs in resource limited regions around the world, lengthening of the human life expectancy and enhanced control efforts of communicable diseases (3, 4). Though NCDs are common in all countries regardless of income level, LMICs have higher NCD-related mortality rates compared to those living in high-income countries (HICs); in fact, 80% of NCD-related deaths occur in LMICs (5). Furthermore, while NCD-related mortality in HICs is associated with older age groups, NCD-related deaths in LMICs are concentrated in younger adults (the 30-69 years old age group). Strikingly, 85% of NCD-related deaths in persons 30-69 years old occur in LMIC (6). These differences between LMICs and HICs demonstrate the profound impact of social determinants of health, such as limited resources, poor health infrastructure and poverty, on global health outcomes related to NCDs and the need to identify region-specific modifiable risk factors for NCDs (7).

Generally, factors such as high blood pressure, smoke exposure, poor glucose control, and obesity are associated with increased risk of NCDs. However, major differences exist between NCD risk factors in HICs compared to LMICs. While diet composition is a standard risk factor for the development of NCDs in both HICs and LMICs, cigarette smoking is a much larger driver of NCDs in HICs compared to LMICs. In contrast, environmental drivers of NCDs (e.g., use of indoor biomass fuel and infectious diseases) are more common in LMICs than HICs. Furthermore, because infectious diseases remain more prevalent in LMICs, NCDs attributed to infections are associated with higher DALYs in LMICs compared to HICs (8).

**Figure 1:**
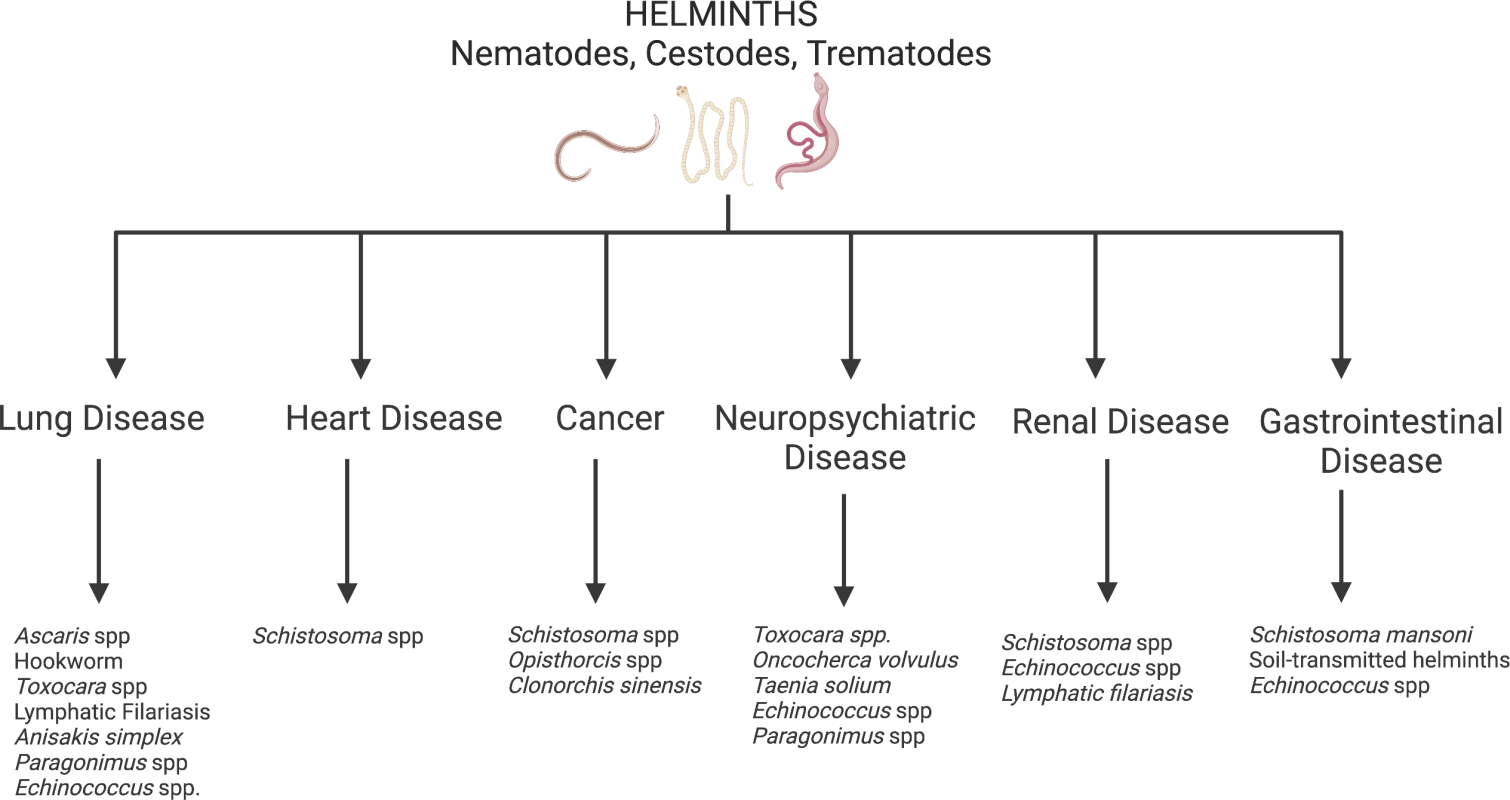
Helminth induced non-communicable diseases. Created with BioRender.com.

Several infectious pathogens are recognized as substantial contributors to the development of various NCDs. Most of these pathogens are more common in LMICs than in HICs. For instance, Hepatitis B virus and Hepatitis C virus are well-known risk factors for liver cirrhosis and cancer. Human Papilloma virus (HPV) and the bacteria *Helicobacter pylori* can also cause cancer (9–11). Many infectious respiratory pathogens are known to result in chronic respiratory disorders including chronic obstructive respiratory disease (COPD; a potential late consequence of tuberculosis, histoplasmosis, etc.) and asthma (which can manifest after childhood Respiratory Syncytial Virus infection) (12, 13). Though less well-known, parasitic infections—from protozoa like *Trypanosoma cruzi* (an infection that manifests as Chagas disease, leading to the development of cardiomyopathy or GI disease in 30% of infected individuals) and *Trypanosoma brucei* (an infection that causes sleeping sickness, leading to chronic neurologic disease in Sub-Saharan Africa) to helminths—can also increase one’s risk for NCDs (14). This review will focus on helminth infections that drive the development of NCDs in endemic LMICs (**Figure 1**). Improving treatment and control of these helminth infections will be an important part of reducing global NCD morbidity and mortality.

## Helminths as a cause of NCDs

Helminths are multicellular parasitic worms that disproportionately infect persons living in poverty-stricken regions of the world. While by far more prevalent in tropical LMICs, helminths prosper in nearly all impoverished regions with climates supportive of the parasitic life cycle (15), including in the southern United States (U.S.). Helminth infections generally do not cause high mortality rates but contribute to high morbidity and subsequent DALYs. Heavy helminth infections can lead to childhood malnutrition, growth restriction and neurocognitive impairment that impact school attainment and work productivity into adulthood (16). Additionally, helminth infections cause clinical manifestations and sequelae that are unique consequences of the individual helminth’s life cycle. For this reason, several helminth infections can lead to NCDs including cardiovascular disease, lung disease, cancer and neuropsychiatric disease (**Figure 1**). On a community level, helminth-induced NCDs could have detrimental consequences on the economic growth in helminth endemic regions, keeping the entire community in poverty.

### Nematodes

#### Soil-transmitted helminths (*Ascaris*, hookworm, *Trichuris*)

Ascariasis (“roundworm”), caused by either *Ascaris lumbricoides* or *Ascaris suum*, is the most common human helminth infection (17). The life cycle of *Ascaris* spp. begins with ingestion of *Ascaris* eggs from the contaminated environment. The larvae hatch in the digestive tract and migrate to the liver followed by the lungs *via* the systemic circulation. In the lungs, the larvae infiltrate the pulmonary parenchyma through the endovasculature and mature into late stage larvae prior to ascending the bronchotracheal tree and returning to the intestines to develop into adult worms (18). The larval migratory phase through the lungs can also cause prolonged mechanical and chemical lung damage leading to functional changes including asthma and chronic obstructive pulmonary disease (COPD). Animal models have shown that pulmonary larval migration causes a coordinated recruitment of eosinophils and neutrophils as well as type 2 T helper cells (Th2) that secrete type-2 cytokines (IL-4, IL-5, IL-13) and Th17 cells. (18, 19). These type-2 and type-17 immune responses inhibit larval development and reduce parasite burden, but lead to extreme allergic airway disease, an asthma phenotype (19, 20). Data from these mouse models have been corroborated in human clinical studies. Previous studies have not only shown an increased risk of asthma (21) but increased asthma severity and need for hospitalization in children exposed to *Ascaris* in endemic regions (22). Together, these data suggest ascariasis is likely a major environmental cause of asthma in endemic regions. Moreover, mice previously infected with *Ascaris* have increased lung volumes, lung compliance and alveolar mean linear intercept (MLI) up to 9 months post infection, representing an emphysematous COPD phenotype. Mechanistic analysis reveals enhanced secretion of matrix metalloproteinase (MMP)-12 from alveolar macrophages, a key mediator in COPD pathology. Though no human clinical studies have yet been done to evaluate the association of COPD with *Ascaris* larval migration, *Ascaris* infection in pigs–a natural *Ascaris* host with similar lung-body weight ratio as humans–can lead to chronic paroxysmal coughing and expiratory dyspnea, suggesting *Ascaris* larval migration can cause chronic lung disease.

Along with the chronic lung disease induced in *Ascaris* mouse models, chronic vascular damage has also been described (23). Studies of mouse lungs 9 months post infection demonstrate pulmonary vascular permeability, erythrocyte extravasation and hemosiderin-laden macrophages, as well as chronic anemia. Anemia is a common clinical manifestation in children with ascariasis classically thought to be secondary to malabsorption of nutrients during the adult intestinal stage (24). However, as mice do not develop patent adult worm infection, the chronic bleeding in the lungs was likely a source of the persistent anemia. After migration through the lungs, *Ascaris* larvae mature and develop into adult worms in the intestines where they live up to 1-2 years. Clinical manifestations of intestinal ascariasis are usually subtle. However, hepatobiliary disease can develop from adult worms invading the biliary tract which can result in biliary strictures, biliary cirrhosis and atrophy of the liver progressing to end stage liver disease (25).

Hookworms, *Ancylostoma duodenale* and *Necator americanus*, infect humans through skin penetration of L3 filariform larvae (infectious stage) found in the soil. L3 larvae travel in the circulatory system to the lungs and enter the alveoli, transcend the trachea and are swallowed back to the gastrointestinal tract, where they molt into L4 larvae and develop into adult worms (26, 27). While there are no clinical studies suggesting hookworm infection is a risk factor for asthma (28, 29), a mouse model of hookworm (using *Nippostrongylus brasiliensis*) found larval migration through the host lungs and mucosal damage increased expression of Trefil factor 2 (TFF2), a central effector molecule in asthma. TFF2 orchestrated chronic lung repair but drove IL-13 associated allergic inflammation and airway hyperreactivity while promoting lung fibrosis after *N. brasiliensis* larval migration (30). In a chronic mouse model of *N. brasiliensis*, larval migration through the lungs induced an emphysematous COPD phenotype (31) as well as persistent hemosiderin-laden macrophages further suggesting the chronic impact of acute hookworm larval migration through the host lungs. The most serious known consequence of hookworm infection is chronic iron-deficiency anemia which can have devastating outcomes in pregnant persons (i.e., pregnancy loss and premature labor) and young children (i.e., cognitive delays) (32, 33). Overwhelming evidence supports that iron deficiency anemia is a direct result of intestinal hookworms infection. Adult hookworms attach to the intestinal mucosa using buccal plates and secret anticoagulant peptides that aid in blood extravasation and digestion utilized for nutrient acquisition by the worm (34). Heavy adult hookworm burden can lead to losses of over 1 mL of blood per day depending on the species of hookworm and the worm burden (35).

Trichuriasis is caused by oral ingestion of *Trichuris trichiura* (“whipworm”) eggs from the contaminated environment (36). After ingestion, larvae hatch in the small intestines and develop into adult worms in the cecum where the anterior end inserts into the colonic mucosa and causes structural changes and localized inflammatory infiltration (37). Children with heavy burden of *Trichuris* can develop colitis similar to inflammatory bowel disease and dysentery syndromes (38, 39). Animal models with chronic trichuriasis have evidence of transmural colonic inflammation and immune profiles dominated by proinflammatory mediators interferon (IFN)-γ, interleukin (IL)-1β, STAT4, tumor necrosis factor (TNF)-α, and IL-6, mimicking both murine models of inflammatory bowel disease and human inflammatory bowel disease (40). Marked elevation of IFN-γ during chronic trichuriasis as well as the production of an IFN-γ homologue by the intestinal helminth have been linked to epithelial dysregulation (41) and progression to chronic *Trichuris* colitis (42). Furthermore, *Trichuris* infection in mice with a genetic predisposition to developing inflammatory bowel disease have accelerated progression of colitis with exaggerated mucosal inflammatory infiltration and cytokine production (43). In addition, clinical studies evaluating children with heavy burden of *Trichuris* infection, indicate colonic pathology with severe, chronic infiltration of inflammatory cells and mucosal destruction consistent with colitis providing supporting evidence that trichuriasis may contribute to human inflammatory bowel disease in parasite-endemic regions (44). Trichuriasis can also be associated with iron-deficiency anemia in children with heavy disease burden resulting from microhemorrhages and blood oozing around the colonic mucosal entry site as seen in *Trichuris* dysentery syndrome (44, 45).

#### Toxocariasis

Toxocariasis is a zoonotic helminth disease of dogs and cats (*Toxocara canis* and *Toxocara cati*) (46, 47). Humans, most commonly young children, become infected with *Toxocara* spp. after ingesting soil contaminated with *Toxocara* spp. eggs, or rarely when ingesting undercooked meat containing larvae. Following ingestion, L3 larvae hatch from the egg in the intestines and migrate to host tissues including the liver, lungs, heart and central nervous system (CNS) (48). Because humans are accidental hosts, *Toxocara* larvae cannot complete their life cycle in humans and die within host viscera overtime, inducing eosinophilic inflammation and granuloma formation (49). Toxocariasis is typically asymptomatic, but larval migration into host tissues can cause syndromes such as visceral larvae migrans (VLM), neurotoxocariasis (NT) and ocular larvae migrans (OLM).

#### NT and epilepsy

Cases of acute NT classically present as eosinophilic meningoencephalitis, myelitis, cerebral vasculitis, or seizures. These clinical manifestations are direct results of larval migration and death within the CNS and the sequalae of the profound inflammatory immune response within tissue (50). Specifically, NT-related epilepsy in children and adults is likely due to increased concentrations of pro-inflammatory cytokines within the brain parenchyma, aberrant neurotransmitter activity and parenchymal scarring (51–53). Murine models of chronic NT demonstrate changes in mRNA expression of key pro-inflammatory cytokines (TNF-*α* and IL-6), inducible nitric oxide synthase, and neurotransmitters (increased norepinephrine and glutamate and decreased GABA, dopamine, and serotonin expression) compared to controls (54–56). Furthermore, *Toxocara* larval migration through tissue induces the formation of eosinophilic granulomas that can cause CNS scarring which become foci of seizure activity. Observed mechanisms of brain injury-measured by increased expression of glial fibrillary acidic protein (GFAP), AβPP, substance P, transforming growth factor β1 (TGF-β1), ubiquitin-proteasome system (UPS), NF-L, S100B, tTG, and p-tau, in mice infected with *Toxocara*–suggest a possible link between NT and chronic neurogenerative (e.g., Alzheimer’s) and neuropsychiatric diseases (57–60). Although, adequately powered clinical studies are needed to make definitive conclusions (61, 62).

#### VLM and asthma

Larval migration through the host lungs is associated with the development of chronic lung disease (63–66). Acute *Toxocara* larval migration and subsequent larval death in the lungs causes wheezing and cough due to airway hyperreactivity and excessive mucous secretion. Beyond acute disease, previous exposure to *Toxocara* is a risk factor for the development of chronic asthma in children (63, 64). *Toxocara* infection in the lungs stimulates innate and type-2 adaptive immune responses, marked by elevated IL-4, IL-5, and IL-13, which results in immunoglobulin class switching to IgE, recruitment and survival of tissue dwelling eosinophils, goblet cell metaplasia and airway hyperreactivity manifesting as asthma. High levels of circulating IgE are capable of binding to mast cells, inciting mast cell degranulation and release of pro-inflammatory mediators further contributing to allergic airway pathology (67–69). Murine models have further demonstrated that *Toxocara* can not only independently cause allergic airway disease but can exacerbate airway inflammation in animals with established allergic airway disease induced by ovalbumin (OVA) sensitization and OVA challenge with overt expression of IL-4, IL-5, and IL-10 (70, 71).

#### OLM and vision loss

OLM most commonly occurs in older children (classically aged 5-10) and adults as a result of *Toxocara* larval migration and death within the eye. Larval death promotes sustained inflammation causing extracellular matrix remodeling and development of eosinophilic granulomas, leading to a wide range of ocular disease including chorioretinitis, vitritis, endophthalmitis, and optic neuropathy as well as retinal detachment leading to blindness (72–75). Animal models of OLM demonstrate that matrix metalloproteinases (MMPs), MMP-2 and MMP-9, and prolonged elevated concentrations of pro-inflammatory cytokines IL-6, IL-8, IL-10, and VEGF aid in recruitment of leukocytes, particularly eosinophils, and break-down fibrin in the posterior chamber extracellular matrix proteins leading to eosinophilic granuloma formation (76, 77). In the U.S. alone, OLM causes approximately 70 cases of blindness annually (78). While reports suggest OLM occurs in 6.6 cases per 100,000 persons, the true global burden of OLM remains unknown (79).

#### Lymphatic filariasis

LF is mosquito-borne disease caused by *Wuchereria bancrofti*, *Brugia malayi*, and *Brugia timori*, though most disease (approximately 90%) is due to *W. bancrofti* (80). Infection occurs when infective stage larvae (L3) are injected into the skin during a mosquito blood meal and travel to the lymphatic vessel where the L3 develop into adult worms (80, 81). Adult worms in the lymphatics release microfilariae into the blood circulation. Pathology is primarily caused by the adult worms, as their presence in the lymphatic vessels can lead to long-term lymphatic inflammation resulting in significant and often disfiguring lymphedema of the lower extremities and other appendages, known as “elephantiasis.” Further, over time, filarial lymphedema is often complicated by bacterial infections of the overlying skin and difficulty with ambulation associated with significant morbidity (80, 81).

Additionally, LF can cause other chronic diseases secondary to the microfilaria stage. Microfilariae that transverse the lungs induce a severe allergic airway disease with airway hyperresponsiveness (manifesting as chronic cough and wheezing), pulmonary and peripheral eosinophilia and pulmonary infiltrates, like clinical asthma, known as pulmonary eosinophilia syndrome (82). Pulmonary function tests reveal pulmonary eosinophilia syndrome cause a restrictive lung disease pattern and may lead to pulmonary fibrosis if left untreated. The pathophysiology is thought to be related to release of filarial antigens that have homology to common allergens such as tPE γ-glutamyl transpeptidase. For instance, *B. malayi* infection may lead to γ-GT specific IgG1 and γ-GT specific IgE antibody expansion (83). Murine models of *B. malayi* have demonstrated that pulmonary eosinophilia syndrome is modulated by type-2 cytokines such as IL-4. Interestingly, this disease phenotype can be suppressed by IL-12, leading to decreased IgE, eosinophilia, and airway hyperresponsiveness, thereby downregulating filaria-induced lung immunopathology (84).

Filarial extrapulmonary pathology may additionally involve the joints and the kidneys. Arthritis (and even vasculitis) attributable to lymphatic filariasis is uncommon (mostly reported in Indian patients with *W. brancrofti*) but may manifest either as oligoarthritis or as polyarticular pseudo-rheumatism (85–88). Symptoms are often unresponsive to non-steroidal anti-inflammatories but improve with anti-filarial treatment (e.g., diethylcarbamazine [DEC]). The pathogenesis is thought to be related to either immune complex deposition or inflammation caused by the presence of the adult worm in the joint space. Regarding the kidneys, patients with filariasis may develop chronic kidney disease manifesting as proteinuria and nephrotic syndrome (89–92). The mechanism is likely driven by immune complex deposition in response to the presence of adult worms and microfilariae. Both the renal tubules and glomeruli may be affected. In an Indian study of 14 patients with filariasis due to *W. bancrofti* and proteinuria, hematuria, or chyluria, six were found to have mesangioproliferative changes, three had inflammatory cell proliferation, and two had endocapillary cell proliferation (93). Immunofluorescence of kidney tissue demonstrated mesangial deposits of IgG alone or in combination with complement 3 (C3) in patients with mesangioproliferative changes and granular deposit of IgG and C3 along the capillary wall in those with endocapillary cell proliferation (93). Another study of patients with filariasis in India due to *B. malayi* indicated that the glomeruli are affected more often than the tubules in symptomatic patients, however noted that proteinuria persisted even after treatment with DEC (94).

#### Onchocerciasis

The filarial worm *Onchocerca volvulus* is well-known to cause vision impairment, primarily in Africa (though disease foci still exist in Yemen, Venezuela, and Brazil) (95). In 2017, more than 1 million people already had vision loss due to the disease (96). Importantly, intracytoplasmic bacteria (*Wolbachia*) often live symbiotically within *O. volvulus*. The pathology of onchocerciasis is likely due to inflammatory responses against both the microfilariae, which circulate in the subcutaneous tissue and lymphatic system and trigger a host inflammatory response (i.e., granuloma formation and eventually fibrosis) when they die, and symbiotic *Wolbachia*. Repeat infections lead to worse cumulative disease. The specific mechanisms that lead to ocular disease remain unclear. Study of tissue sections have demonstrated infiltrates containing plasma cells, eosinophils, and mast cells (97). Degenerating microfilariae may cause punctate keratitis. Inflammation (and possibly an autoantibody formation) (98, 99) related to microfilariae can lead to anterior uveitis and chorioretinitis. Corneal pathology is associated with increased systemic and corneal type-2 cytokines expression, illustrated by *in vivo* studies in which IL-4 gene knockout mice developed less severe or no *O. volvulus*-mediated keratitis (100). Regarding molecular mimicry, *O. volvulus* antigen Ov39 is cross-reactive with the retinal antigen hr44 and induces ocular inflammation in rats (99). Interestingly, corneal inflammation is not induced by extracts derived from *O. volvulus* depleted of *Wolbachia* (101), and may be related to expression of adaptor molecules such as TIRAP/Mal (101) and myeloid differentiation factor 88 (102), which is a necessary part of some toll like receptor (TLR) signaling pathways.

*O. volvulus* may also contribute to a form of incompletely described, but likely progressive epilepsy known as “nodding disease” (103, 104). Nodding disease has been primarily recognized among children in east Africa (particularly Tanzania, Sudan, and Uganda) though outbreaks over the last decade are reported from Uganda, Liberia, Tanzania, the Democratic Republic of Congo, and southern Sudan. Nodding disease classically manifests as episodes during which the head bobs forward repeatedly for several minutes and the individual may seem unresponsive; these episodes are sometimes associated with generalized tonic-clonic and/or absence seizures. Nodding syndrome may progress to significant cognitive disability and, eventually, death (105). No specific cerebrospinal fluid or neuroimaging abnormalities are yet associated with the disease, though some individuals are noted to have significant atrophy of the hippocampal and glia matter (106). The association between *O. volvulus* and nodding disease came about after one Ugandan study suggested a higher rate of epilepsy in communities with higher *O. volvulus* prevalence (107), however, the underlying biological mechanism for such an association remains unclear (108–110). One possibility under active investigation is that nodding syndrome may be due to autoantibodies (e.g., to leiomodin-1) produced in response to *O. volvulus* infection (111).

### Trematodes

#### Schistosomiasis

Three *Schistosoma* species cause most intestinal (*S. japonicum* and *S. mansoni*) and urogenital disease (*S. haematobium*). The host immune response to *Schistosoma* species eggs is responsible for the clinical manifestations of the disease syndromes. Paired adult worms reside in small veins (of the lower urinary tract for *S. haematobium* and of the mesenteric plexus for *S. japonicum*) and release *Schistosoma* species eggs which migrate through associated organs, thereby causing considerable irritation, prolonged inflammation, and granuloma/fibrosis development (112, 113). Schistosomiasis egg migration and the subsequent chronic immune activation can have a wide range of effects on end-organ disease.

#### Schistosoma-induced renal disease

Renal disease, ranging from asymptomatic to end-stage renal disease (ESRD), is a result of direct egg induced inflammation or *Schistosoma* antigen - immune complex formation within the kidney most commonly seen in *S. mansoni* hepatosplenic disease (114). In a Brazilian longitudinal study of 24 patients with schistosomiasis (likely *S. mansoni* as this is the major species found in Brazil), fifteen (68.1%) had related hepato-splenic disease, thirteen (54.1%) had nephrotic-nephritic syndrome, twenty (83.3%) had hematuria and 18 (75.0%) had hypertension. After nearly 60 months of follow up, nine patients developed ESRD (115). Approximately 5-6% of patients with hepatosplenic schistosomiasis develop glomerular involvement (114). The most common schistosomiasis glomerular disease is mesangial proliferative glomerulonephritis followed by membranoproliferative glomerulonephritis and less commonly focal and segmental glomerulosclerosis, exudative glomerulonephritis and amyloidsis (114, 116). Additionally, urogenital schistosomiasis from *S. haematobium* can cause lower urinary track fibrosis and calcification which can lead to renal outlet obstruction, ureter reflux, interstitial nephritis and ESRD (116).

#### Schistosomiasis-induced liver disease

*S. mansoni*’s predilection for the venous portal-mesenteric system can result in liver fibrosis, portal hypertension as well as end-stage liver disease. In hepatosplenic schistosomiasis, portal hypertension occurs from an eosinophilic granulomatous reaction to *Schistosoma* egg deposited in presinusoidal portal venules causing presinusoidal hepatic fibrosis typically with preserved liver function (117). Imaging of the liver often shows splenomegaly and calcified eggs along enhanced portal tracks in the liver by CT scan (118). The consequences of portal hypertension include gastric bleeding, such as esophageal varices, which is responsible for an estimated 200,000 deaths annually in sub-Sahara Africa, and ascites (119). Polarized Th2 immune responses during hepatosplenic schistosomiasis not only promotes liver fibrosis but also impairs Th1 anti-viral immunity. Co-infection with HBV and/or HCV, common in schistosomiasis-endemic regions, can accelerate liver pathology particularly advancing viral-induced hepatocellular carcinoma and liver failure (120, 121).

#### Schistosomiasis-associated pulmonary hypertension (Sch-PH)

Sch-PH is believed to be a leading cause of pulmonary hypertension in *Schistosoma* endemic regions around the world and can result in right-sided heart failure (122). Sch-PH is most commonly associated with chronic hepato-splenic schistosomiasis as a result of *Schistosoma mansoni* infection. Approximately 5-10% of patients with hepato-splenic schistosomiasis will develop Sch-PH which can result in devastating cardiovascular disease including end-stage right ventricular heart failure (123, 124). The immunopathogenesis of Sch-PH is likely multifactorial. Hepato-splenic disease causing portopulmonary hypertension may occur due to the underlying liver disease or from *Schistosoma* egg embolization and inflammation induced vasculopathy with medial thickening, intimal remodeling and formation of granulomas and fibrosis (125).

#### Schistosomiasis and malignancy

*S. haematobium* and *S. japonicum* are both designated biological human carcinogens by the International Agency for Research on Cancer (126).

Urogenital schistosomiasis due to *S. haematobium* is common in endemic areas and complicated by the development of bladder cancer. The incidence of urogenital schistosomiasis-associated squamous cell bladder carcinoma is estimated at 3-4 cases per 100,000 (127). Several mechanisms have been implicated in the oncogenesis of *Schistosoma haematobium* infection (128): 1) *Schistosoma* antigen can increase proliferation and longevity of urothelium cells (129) 2) Elevated p53 levels have been documented in both pre-malignant and malignant lesions associated with schistosomiasis (130, 131) 3) Oncogenic mutation of the KRAS gene can be induced in urothelium exposed to whole parasite extract (132) 4) Soluble *Schistosoma* egg antigens (SEA) increase proliferation and oxidative stress and decrease apoptosis (133) 5) Repeated deposition of eggs in the bladder wall and migration of the eggs through the urothelium results in chronic inflammatory infiltrate (134) and parasite-induced oxygen derived free radicals, genetic mutations and the production of carcinogenic compounds (131, 133, 135) as well as 6) Epigenetic changes *via* hypermethylation of the host genome (136). Of note, *S. haematobium* may contribute to other types of cancer as well. An autopsy study from the Central Pathology Institute of Baghdad, Iraq found that, between 1939-1952, of 2276 autopsies, 174 had carcinoma and 113 had *S. haematobium* involvement of the bladder (137). *S. haematobium* was present in 3 with liver cancer, 7 with bladder cancer, 3 with prostate/genitalia cancer, 2 with intestinal/rectal cancer, and 2 with undescribed cancer types. Particularly in the case of liver cancer, co-infection with *S. haematobium* plus another carcinogenic organism, such as the hepatitis B virus, may amplify *S. haematobium*’s carcinogenic potential (138). Additionally, *S. haematobium* can work in concert with HPV to promote the development of cervical cancer. Proposed mechanisms include *S. haematobium* induced mechanical damage to the cervical epithelium and *S. haematobium* induced local immune modulation creating a niche for HPV proliferation (139, 140).

A less common *Schistosoma* species, *S. japonicum*, is more often associated with liver cancer and colorectal cancer (141), although this is mostly based on epidemiologic associations (142–144) and pathology data (145, 146). *S. japonicum* eggs, retained in the intestinal wall, cause prolonged irritation that can result in fibrosis, mucosal hyperplasia, polyp development, and adenocarcinoma formation (147, 148). Additionally, an “egg embolism” can occur leading to pathology in the liver and other organs (149). A pathology study evaluating the association between *S. japonicum* and liver cancer in 4,611 necropsies revealed 227 cases of hepatocellular carcinoma (HCC); 24 (10.6% of these) were associated with *Schistosoma japonica*. Importantly, 27% of these cases had a positive Hepatitis B surface antigen, indicating the possibility that multiple types of co-infection may synergistically contribute to carcinogenesis (150). *S. japonicum* likely induces multiple mechanisms that contribute to malignant transformation of colonic and/or liver tissue. These mechanisms include chronic inflammation (147, 151), carcinogenic molecules derived from *S. japonicum* itself (152, 153), immunomodulation (154, 155), and oncogenic mutations (156).

#### Liver flukes

Two trematodes, or liver flukes, are associated with malignancy (*Clonorchis sinensis* and *Opisthorchis viverini* with cholangiocarcinoma) and, similar to schistosomiasis, are classified as biological human carcinogens by the International Agency for Research on Cancer (126, 157, 158). Liver flukes’ relative contributions to carcinogenesis are likely related to the length and severity of infection, the host’s immune status, and other environmental and host genetic factors (159). Both *Clonorchis sinensis* and *Opisthorchis viverrini* are food-borne trematodes found in East Asia; *C. sinensis* is endemic to southern China, Korea, eastern Russia, and northern Vietnam, whereas *O. viverrini* is endemic to Thailand, Lao People’s Democratic Republic, Cambodia, and central Vietnam. Though fewer than 10% of people with liver fluke infections will develop cholangiocarcinoma, the incidence of cholangiocarcinoma is significant in regions of high *C. sinensis* or *O. viverrini* prevalence. For example, the incidence of *O. viverrini*-associated cholangiocarcinoma is approximately 98 per 100,000 people in the highly endemic region of the Thai province of Khon Kaen, where the prevalence of *O. viverrini* infection ranges from 2-70% (160). A Korean study published in 1996 showed that 33% of the cholangiocarcinoma cases evaluated were positive for *C. sinensis* by stool examination (161). Adult flukes can inhabit the biliary track for decades and cause recurrent pyogenic cholangitis. This repeated and/or prolonged inflammation of the biliary tree may contribute to later development of chronic biliary track disease including cholangiocarcinoma, however the exact mechanism(s) by which the liver flukes contribute to carcinogenesis are not yet fully elucidated. Likely liver fluke infection generates multiple mechanisms leading to carcinogenesis, including 1) Mechanical damage from physical contact of the biliary epithelium with the parasite, 2) Inflammatory pathology related to the host-parasite immune response, and 3) Prior to chemical damage from fluke excretory-secretory products (ESPs) (158, 162, 163). A sizeable body of literature exists detailing the carcinogenic potential of ESPs. Fluke ESPs can induce proliferation (164, 165), apoptosis (166, 167), chromatin remodeling (166), and inflammation of biliary epithelial cells (168). Resulting repetitive insults to the biliary epithelium may lead to hyperplasia and adenomatous changes with subsequent malignant transformation (158).

#### Paragonimiasis

Paragonimiasis, the lung fluke, results from ingesting metacercariae in raw or undercooked crab or crayfish. The metacercariae encyst in the duodenum, travel through the intestinal wall into the peritoneal cavity, abdominal wall and diaphragm transversing into the lungs and subsequently encapsulating into adult flukes within lung parenchyma (169). During active infection, paragonimiasis can mimic the radiographic appearance and clinical manifestations of pulmonary tuberculosis or even lung cancer, including pleural disease, solitary nodules and cavitary lesions, commonly presenting with chronic cough, chest pain and hemoptysis (170–172). However chronic lung pathology can also occur from pleuropulmonary paragonimiasis. Infection left untreated or repetitive infection with *Paragonimus* spp, most commonly *Paragonimus westermani*, can lead to the development of bronchiectasis and chronic bronchitis (173, 174). The pathophysiology of *Paragonimus* spp. associated chronic lung disease remains unknown. More robust animal models and human clinical studies are needed.

Although rare (occurring in approximately 0.8% cases of paragonimiasis), aberrant migration of *Paragonimus* spp to the brain can also lead to the development of hemorrhagic stroke and epilepsy. In cases of ectopic paragonimiasis, 30-60% occur in the brain and most commonly occurs in children (175). Adult worms migrate through the perivascular connective tissue around the jugular vein and carotid artery into the posterior circulation *via* the skull base foramina leading to mechanical damage secondary to parasitic migratory tracts and formation of eosinophilic granulomas with central necrosis and charcot-leyden crystals (176–178). Early disease typically presents as meningoencephalitis, vasculitis and necrotizing granulomas, manifesting clinically as epilepsy and hemiplegia. Cerebral paragonimiasis can also cause pseudoaneurysms that are often misdiagnosed as noninfectious vascular malformation in children. In 17 patients with cerebral paragonimiasis and hemorrhagic strokes 35% had evidence of pseudoaneurysm and pseudoaneurysm rupture (179). In children with chronic cerebral paragonimiasis, CNS investigation reveals perivascular granulomas formation and calcification and associated cortical and subcortical atrophy (175). In a case series of 14 children with cerebral paragonimiasis, intracranial hemorrhage and eosinophilic granulomas were commonly identified. Despite targeted therapy, two of the 14 children had persistent hemiplegia on long-term follow-up secondary to sequelae of hemorrhagic strokes (180). In addition to epilepsy and paralysis, children with cerebral paragonimiasis can also have long-term behavioral changes. If treated early with antiparasitic therapy, seizures generally improve. However, dizziness, memory loss, personality changes, and loss of fine motor function, often do not completely resolve (181).

### Cestodes

#### Neurocysticercosis (*Taenia solium*)

*Taenia solium* (the pork tapeworm) can cause intestinal disease (“taeniasis”), which is acquired after ingestion of *T. solium* larvae (“cysticerci”) *via* infected pork or cysticercosis, due to inadvertent consumption of *T. solium* eggs *via* exposure to a person or pig with taeniasis (182, 183). Cysticercosis can develop once *T. solium* eggs hatch into larvae in the intestine, migrate to various host organ systems, and develop into cystic larvae within the tissue. Neurocysticercosis (NCC) is the most consequential form of cysticercosis and occurs when cysticerci develop in the CNS (including the brain, eyes, and spinal cord). NCC is a leading cause of adult-onset epilepsy worldwide (184). Most of the clinical manifestations seen in NCC are related to the host immune response to the parasite, and are dependent on the number, stage, and size of cysticerci. For instance, nearly 80% of patients diagnosed with NCC report at least one lifetime seizure (185). Seizures typically are seen in patients who have parenchymal NCC (186). They are triggered by disruption of the brain parenchyma and therefore neuronal signaling caused by certain stages of *T. solium* cysts, specifically as cysts naturally progress from viable to a final calcified stage (187). Viable cysts are often able to evade the immune response (187–189), thus it is the inflammatory reaction to degenerating cysts (which have lost their ability to modulate the host immune system) and mechanical obstruction of normal neuronal pathways caused by residual calcifications from old cystic lesions that provoke seizures (190). Further, the degree of cyst-induced inflammation may be directly related to frequency of seizure recurrence (191). Studies of human brain tissue sections with NCC suggested that the initial immune response (prior to peri-cyst granuloma formation) is characterized by innate and Th1 cells and cytokines, including natural killer cells, macrophages, T cells, and interleukin (IL)-12 (187). Subsequently, as granulomas develop around degenerating cysts, a chronic immune reaction develops, characterized by both type-1 and type-2 immune responses (192). Additionally, symptomatic NCC patients may produce lymphocytes primed towards Th1 responses (193) and have distinct TLR polymorphisms (194). Calcified NCC specifically has been associated with not only unique TLRs but also higher serum levels of matrix metalloproteinases (MMP)-9; the same study found MMP-9 to be associated with seizure recurrence (195).

While most patients diagnosed with NCC have parenchymal disease (and calcified parenchymal disease is more common than viable parenchymal cysts), a rare but important subset of NCC is extra-parenchymal disease (186). Extra-parenchymal disease is defined by cystic lesions that develop in the ventricles, subarachnoid space, spine, or retina. Ventricle and subarachnoid cysts can cause symptoms *via* mass effect on surrounding tissues, the host inflammatory response to the cyst tissue can trigger aseptic meningitis, and both the cysts themselves and scarring from accompanying inflammation can cause obstructive and/or communicating hydrocephalus that often requires invasive intervention *via* placement of a ventriculo-peritoneal shunt or surgical removal of cysts (196). Cerebrospinal fluid from patients with subarachnoid disease seem to have significantly elevated levels of type-1 and type-2 cytokines compared to patients with parenchymal disease (197, 198).

#### Echinococcosis

Cystic echinococcosis (*E. granulosus*) and alveolar echinococcosis (*E. multilocularis*) results from ingestion of *Echinococcus* spp eggs in contaminated soil (199–201). From the intestines the eggs hatch releasing oncospheres that penetrate the intestinal wall and travel classically to the liver or lungs and, aberrantly, to other organs like the brain where they develop into thin-walled cysts. Patients with *Echinococcus* cysts are typically asymptomatic until the lesions create a mass effect on surrounding tissue or rupture causing a systemic inflammatory response (201). The clinical course of echinococcosis in different organ compartments is associated with a wide spectrum of complications that can lead to chronic disease in the liver, lungs, brain and kidneys (201, 202). In a cohort of 506 patients with cystic echinococcosis, 204 developed long-term complications as a result of their illness (202).

Patients with liver lesions, from either Cystic *Echinococcus* or Alveolar *Echinococcus*, have high risk of hepatic complications. Approximately 1/3^rd^ of all patients with hepatic disease will develop a long-term complication of the hepatobiliary system including biliary track fistulas, biliary cirrhosis, cholangitis, pancreatitis and portal hypertension with associated gastrointestinal bleeding (201, 203). Mass effect from hepatic cysts cause increased pressure on hepatic and biliary tissue leading to compression and necrosis of adjacent tissues. Bile duct damage, rupture and subsequent development of biliary fistulas are thus common. The mass effect of hepatic cysts can also reduce portal vein inflow causing portal vein thrombosis and portal hypertension as well as compression and displacement of hepatic veins leading to Budd-Chiari syndrome. Liver pathology in patients with *E. multilocularis* hepatobiliary cysts were found to have periportal fibrosis, perilobular fibrosis and amyloid deposition (204). Pulmonary cysts can also cause mass effect on surrounding mediastinal structures, however clinical manifestations most commonly result from acute rupture of pulmonary cysts, release of immunogenic antigens from the cystic fluid, and exaggerated type-2 immune responses leading to an asthma-like syndrome of wheezing and coughing (201). Furthermore, some mouse models have shown that the presence of *Echinococcus* antigens may exacerbate established allergic airway disease through increased type-2 cytokine signaling resulting in histopathologic changes consistent with asthmatic disease (205). Although rare, occurring in approximately 1% of all cases of *E. multilocularis*, cerebral echinococcosis is associated with high mortality, with a 10-year survival rate of 28.4%. The presence of *E. multilocularis* cystic fluid can have cytotoxic effects on the surrounding cerebral parenchyma resulting in inflammatory recruitment and tissue necrosis (206). Of those with cerebral echinococcosis that survive, nearly 30% will have long-term neurologic sequalae including epilepsy, vision loss and hemiplegia (207–209). Several case reports and case series suggest that echinococcosis from either *E. granulosus* or *E. multilocularis* can be associated with kidney disease. The mechanism of *Echinococcus* renal disease is thought to be immune-complex mediated leading to tubulointerstial nephritis, glomerulonephritis (minimal change disease, mesangioproliferative) and nephrotic syndromes (210). In a case series of patients with hepatic cysts with associated proteinuria, kidney biopsy demonstrated disease was driven by hydatid antigen and the development of immune-mediated glomerulonephritis (211–213). As the literature related to extra-hepatic echinococcosis is limited to case reports and cases series, more in depth studies are needed to determine the mechanisms of *Echinococcus* driven extra-hepatic chronic diseases.

## Conclusions

Helminths, which disproportionately affect persons living in poverty within LMICs, represent a major driver of morbidity on a global scale. Their role in driving NCDs including chronic lung disease, cancer, cardiovascular disease and inflammatory bowel disease are plausible but more robust mechanistic studies and human clinical trials are required to draw definitive conclusions (**Table 1**). These studies are necessary to aid in uncovering risk factors associated with the rising incidence and prevalence associated with NCDs in LMIC and other helminth-endemic regions around the world. Understanding this connection between helminths and NCDs will aid in targeted public health programs to reduce helminth-induced NCDs and reduce the high rates of morbidity in these regions. In the mean-time focus on helminth prevention and control efforts are critical. Access to mass drug treatment programs, water, sanitation, and hygiene interventions and health education programs for high-risk populations, especially children and pregnant persons, will result in reduced worm burdens and subsequently reduced morbidity. Elimination efforts should also remain a public health priority as the complex mechanisms of helminth induced NCDs are being evaluated.

**Table 1:** Proposed mechanisms of helminth induced non-communicable diseases.

Helminths	Non-communicable disease	Proposed Mechanism of disease	References
**Nematodes**			
*Ascaris* spp.	Larval migratory stage: anemia, asthma, chronic obstructive pulmonary disease Adult intestinal stage: anemia, biliary stenosis, end-stage liver disease	-Chemical damage from excretory secretory products (ESPs) with immunogenic proteins during larval migration -Mechanical damage from direct larval migration -Adult intestinal worm obstructing the biliary tract	(15, 18, 20, 23, 25, 214)
Hookworm (*Ancylostoma duodenale*; *Necator americanus*)	Larval migratory stage: chronic obstructive pulmonary disease Adult intestinal stage: anemia	-Chemical damage from ESPs with immunogenic proteins during larval migration -Mechanical damage from direct larval migration -Adult intestinal worm extravasation of blood	(30–34)
*Trichuris trichiura*	Adult intestinal stage: inflammatory bowel disease, anemia	-Insertion of anterior end into intestinal mucosa inducing local inflammation	(40, 41, 43, 45, 215)
*Toxocara cani/cati*	Larval migratory stage: Asthma, epilepsy, neurodegenerative diseases, neurobehavioral diseases, vision loss/blindness	-Larval migration and death in viscera causing eosinophilic infiltration and granulomas	(47, 51–53, 59, 61, 64, 68, 70, 75, 76, 216)
*Anisakis simplex*	Asthma, urticaria	-Cross-reactivity of *A. simplex* antigen with common allergens -High levels of *A. simplex* specific IgE	(217–219)
*Lymphatic Filariasis (Wuchereria bancrofti, Brugia malayi, Brugia timori)*	Lymphangitis and resulting complications related to chronic lymphedema (e.g., elephantiasis) and/or urogenital disease including kidney disease; less commonly, arthritis Microfilariae: pulmonary eosinophilia syndrome, renal disease	-Adult worms cause inflammation in the afferent lymphatic channels, leading to chronic obstructive changes -Renal disease likely due to immune complex deposition, among other mechanisms -Lung disease due to filarial antigens that have homology to common allergens, IL-4 mediated	(81, 83, 86, 87, 94)
*Loa loa*	Chronic kidney disease; less commonly, arthritis	-Etiology not completely elucidated	(88, 88, 220, 221)
*Onchocerca volvulus*	Vision impairment and dermatologic disease, progressive epilepsy	-Tissue damage due to inflammatory response to microfilariae and *Wolbachia*; an additional autoimmune component is suspected	(98–100, 107, 108, 110)
**Trematodes**			
*Schistosoma*	Malignancy (bladder, liver, colon, cervical), pulmonary hypertension and end-stage heart disease, portal hypertension, chronic genito-urinary track disease (e.g., glomerular damage leading to hypertension, nephritis, nephrotic syndrome, end-stage renal disease)	-*Oncogenic mutations in* p53 and KRAS the urothelium -*Schistosoma egg* antigen increases proliferation and longevity of host cells. -*Schistosoma egg* induce chronic inflammation, fibrosis, granuloma formation	(114, 117, 122, 129, 130, 132, 141, 142, 144, 222)
*Clonorchis* *Opisthorchis*	Malignancy (cholangiocarcinoma)	-Mechanical damage from physical contact of the biliary epithelium with the parasite -Inflammatory pathology related to the host-parasite immune response, and chemical damage from fluke ESPs.	(158, 161, 163, 166, 167)
*Paragonimus*	-Bronchiectasis, chronic bronchitis -Epilepsy, stroke	-mechanical damage secondary to parasitic migratory tracts and eosinophilic granulomas	(174, 176–178)
**Cestodes**			
*Taenia solium*	When manifested as neurocysticercosis: epilepsy, obstructive and/or communicating hydrocephalus	-Host immune response to degenerating cysts in the central nervous system -Mechanical obstruction of normal neuronal pathways caused by residual calcifications from old cystic lesions	(184, 185, 187, 192, 197)
*Echinococcus*	-Hepatobiliary: biliary track fistulas, biliary cirrhosis, cholangitis, pancreatitis, and portal hypertension with associated gastrointestinal bleeding -Pulmonary: asthma -Cerebral: epilepsy, stroke -Renal: tubulointerstial nephritis, glomerulonephritis (minimal change disease, mesangioproliferative) and nephrotic syndromes.	-Mass effect from hepatic cysts causes increased pressure on hepatic and biliary tissue -Acute rupture of pulmonary cysts, release of immunogenic antigens from the cystic fluid, and exaggerated Th2 immune response -Cytotoxic effects on the surrounding cerebral parenchyma resulting in inflammatory recruitment and tissue necrosis -Immune-mediated	(201, 206, 210, 223)

## Author contributions

YW and JW conceptualized, wrote and edited the manuscript. MD, SA, CS-R, and EC wrote and edited the manuscript. All authors contributed to the article and approved the submitted version.

## Funding

This work was supported by NIH NIAID K23 K23AI168583-01 and NIH NIAID K08 AI143968-01.

## Conflict of interest

The authors declare that the research was conducted in the absence of any commercial or financial relationships that could be construed as a potential conflict of interest.

## Publisher’s note

All claims expressed in this article are solely those of the authors and do not necessarily represent those of their affiliated organizations, or those of the publisher, the editors and the reviewers. Any product that may be evaluated in this article, or claim that may be made by its manufacturer, is not guaranteed or endorsed by the publisher.
